# Moving the debate forward: interculturalism’s contribution to multiculturalism

**DOI:** 10.1186/s40878-018-0078-2

**Published:** 2018-05-17

**Authors:** François Boucher, Jocelyn Maclure

**Affiliations:** 1 Centre for Ethics, Social and Political Philosophy, KU Leuven, Institute of Philosophy, Kardinaal Mercierplein 2, 3000 Leuven, Belgium; 2 Faculty of Philosophy, Université Laval, Pavillon Félix-Antoine-Savard, 2325, rue des Bibliothèques, Université Laval, Québec, QC G1V 0A6 Canada

**Keywords:** Contact hypothesis, Intercultural dialogue, Integration, Interculturalism, Multiculturalism, Ricard Zapata-Barrero, Tariq Modood

## Abstract

In this article, we compare Ricard Zappata-Barrero’s interculturalism with Tariq Modood’s multiculturalism. We will discuss the relation between distinct elements that compose both positions. We examine how recent discussions on interculturalism have the potential to contribute to theories of multiculturalism without undermining their core principles. Our position is close to that of Modood’s as he has already carefully tried to incorporate interculturalist insights into his own multiculturalism. Yet we provide a raise a few questions regarding Modood’s treatment of the relation between multiculturalism and interculturalism. After summarizing each author’s potion (I), we will comment on the following set of relations between their basic elements: (II) The relation between intercultural contact and intercultural dialogue; (III) The relation between contact at the local level and the societal/state level; (IV) The relation between group-specific measures, intercultural contact and mainstreaming.

## Introduction

Zapata-Barrero (2017) offers an accurate and nuanced depiction of the dominant political narrative of the rise and fall of interculturalism and multiculturalism. The diagnostic he poses has three moments: the fall of multiculturalism, the rise of civic integration and the rise of interculturalism. After enjoying tremendous popularity in the 1990s, multiculturalism came under attack from both ends of the political spectrum in the 2000s. The discontent with multiculturalism reached its peak in 2010 when its death was proclaimed by European leaders (Cameron, Merkel and Sarkozy). The dominant discourse claims that multiculturalism celebrates difference but not unity, that it grants rights to immigrants without imposing duties to integrate to the host society, and that it leads to the fragmentation of society by enabling them to live in parallel micro-societies. Two political reactions came out of the proclaimed fall of multiculturalism. On the one hand, a nationalist discourse of civic integration emerged, promoting a duty-based approach according to which government should enforce the obligation to integrate and to embrace the values of the host societies, mostly through citizenship tests (Goodman, 2010; Joppke, 2007; Joppke, 2012; Joppke, 2017). On the other hand, interculturalism emerged as an allegedly more diversity-friendly discourse refusing both the multicultural fragmentation of society and the nationalistic and coercive overtone of civic integration.

Although we agree with this picture of the state of the political discourse on multiculturalism today, we embrace Modood’s (2017) claim that this widespread view is not critical enough of the dominant narratives on multiculturalism. The discourse on the death of multiculturalism, and the accompanying celebration of either civic integration or interculturalism, distorts and misrepresents multiculturalism both in theory and in practice. On the one hand, interculturalism and multiculturalism, at least in their liberal-egalitarian variants, share a common normative core at the level of political morality, as they both reject cultural assimilationism and promote the respect of ethnocultural practices compatible with basic liberal democratic principles (Maclure, 2010). In recent years, theoretical accounts multiculturalism have mostly adopted a civic variant of multiculturalism, one that emphasize cross-cultural interactions and dialogue as a way of creating new forms of belonging in diverse societies (Maclure, 2010, p. 41). On the other hand, multiculturalism is not against promoting integration, it seeks to provide immigrants with fair terms of integration (Balint & de Latour, 2013; Kymlicka, 1995). As such, it not incompatible with liberal measures of civic integration, although it severely condemns illiberal projects of civic integration as assimilationist projects in disguise (Kymlicka, 2012, pp. 15–16).

Nonetheless, in comparing both papers, it appears that important differences arise between Zapata-Barrero’s (2017) interculturalism and Modood’s (2017) multiculturalism. To comment on those two important contributions, we propose to do a bit of chemistry. We will comment on the relation between the distinct *elements* that form the *composite substance* of both positions in order to find our way to a mixture optimally adapted to the current predicament of immigration societies. We believe such mixture has a similar taste than Modood’s brew as he has already carefully tried to incorporate interculturalist insights into his own multiculturalism, yet some fermentation is still needed. After summarizing each author’s potion (I), we will comment on the following set of relations between basic ingredients: (II) The relation between intercultural contact and intercultural dialogue; (III) The relation between contact at the local level and the societal/state level; (IV) The relation between group-specific measures, intercultural contact and mainstreaming.

### I interculturalism and multiculturalism: distinction and incorporation

According to Zapata-Barrero (2017), the three distinctive features or basic elements of interculturalism as a policy paradigm distinct from both civic integration and multiculturalism are (1) that it promotes contacts and interactions between individuals with different ethnocultural backgrounds, (2) that it focuses on the local level and (3) that it relies on mainstreaming strategies. Interculturalism encourages “contact between people from different backgrounds” (p. 7–8). Here, ‘contact’ refers to face-to-face interactions between individuals of different ethnocultural groups (p. 14). Those interactions are distinct events located in space and time involving real persons and therefore, as we will explain later, the notion of intercultural *contact* is distinct from that of intercultural *dialogue*. Moreover, Zapata-Barrero insists that contacts cannot be obtained by coercive means (p. 9). It is because of this emphasis on contacts that interculturalism operates at the local level, that is, the level where face-to-face interactions happen. In contrast, both multiculturalism and civic integration, with their respective focus on rights and duties, operate exclusively at the state level (p. 13). According to him, interculturalism ignores the dead-end task of specifying the rights and duties of immigrants and turns to the more productive one of promoting contact zones and shared spaces within cities. Finally, he adds that both civic integration and multiculturalism design policies which target immigrant groups as their recipients, thereby creating a distinction between “us”, the natives, and them, the newcomers. The former singles out immigrants as the bearers of duties to integrate and as subjects to citizenship tests (Goodman, 2010), the latter targets immigrants as the bearers of group-specific rights (Kymlicka, 1995). Interculturalism aims to avoid reinforcing this us/them distinction by relying exclusively on mainstreaming policies, that is, on measures that do not specifically target immigrant groups but rather the whole population (p. 15–16).^1^

Modood (2017) disagrees with the claim that there is a sharp contrast between interculturalism and multiculturalism. He notes that within the fields of political theory and political philosophy, researchers have for long emphasized the role of intercultural dialogue as well as the qualified importance and recognition of the majority. To oppose interculturalists such as Cantle (2016) and Zapata-Barrero who claims that their views are radically new because they emphasize intercultural interactions, he carefully draws on the scientific literature on multiculturalism to show, convincingly, that most canonical texts in this field, Taylor’s (1994) *The Politics of Recognition*, Young’s (1990) *Justice and the Politics of Difference*, Parekh’s (2000) *Rethinking Multiculturalism*, Tully’s (1995) *Strange Multiplicity* and his own *Multiculturalism: A Civic Idea* (Modood, 2007) embrace dialogue between different cultural traditions as a method to arrive at fundamental political principles. To counter the claim that multiculturalists are insensitive to the legitimate goals and aspirations of the ethnocultural majority—a claim championed by Bouchard (2015)—he explains how the recognition of the majority in his theory is compatible with group recognition. To show this, he claims that symbolic recognition of minorities can be done in an even-handed fashion, without removing existing privileges for the majority, and that instead of removing existing religious instruction for the majority religion, we should offer religious instruction for religious minorities as well.^2^

The picture of multiculturalism drawn by Modood (2017) clearly differs from the caricature promoted by the post-multiculturalism discourse, but it also differs sensibly from Zapata-Barrero’s (2017) interculturalism, for whom the three pillars of interculturalism are the promotion of contacts between individuals with different cultural attachment, the rejection of a state-centric policy paradigm and the corresponding promotion of the role of cities, and the promotion of mainstreaming as opposed to group-targeted measures. Modood’s view in comparison is still, for the most part, state-centric, as we explain in the next section (and this is not necessarily a problem as we will explain in section III) and emphasize group-targeted measures and group recognition. Modood’s multiculturalism is thus much closer to Bouchard’s majoritarian interculturalism than it is to Zapata-Barrero’s “interactionist” variant (Rocher & White, 2014).^3^

### II contact and dialogue

The notion of intercultural contact or interaction is conceptually distinct from the broader notion of intercultural dialogue, which normally refers to the societal process by which ideas, values and practices emerging from different traditions adjust to one another and negotiate their expression within a shared public sphere. Intercultural dialogue refers to the meeting of ideas, values, symbols and arguments, whilst intercultural contact refers to the meeting of real people in specific physical places. Of course, the two notions are not logically mutually exclusive, but they do not imply one another. A specific intercultural contact between two individuals from different backgrounds may or not lead to an intercultural dialogue—it can for instance consist of a discussion about the weather occurring at the park—and intercultural dialogue may or not be a process involving widespread intercultural face-to-face contacts in daily life—it can for instance consists of a series of written communications in newspapers or academic journals. Most importantly, emphasizing the value of one or the other may command different policies. The importance of intercultural dialogue is likely to require that government be more sensitive to the values and practices of minority groups expressed by their elites in various formal settings (courts, official public consultations, legislative assemblies and so on), whereas the importance of intercultural contacts is likely to require policies of urban planning emphasizing the creation of urban spaces conductive to positive intercultural encounters.

Modood’s (2017) contribution highlights that the multiculturalism literature in political theory relies on intercultural dialogue as a method to build normative political principles:

Multiculturalists have rejected abstract reasoning by a sole reasoner or identical identity–less individuals in favour of dialogue. They assume that the context for politics is already thoroughly imbued with dominant ways of thinking and doing—with cultural orientations such as national history and language, with religious and/or secular perspectives, with institutional norms and so on—and that these contextual factors cannot be abstracted out so as to identify a set of principles of justice independent of cultural interpretations. (p. 5).

This, however, in addition to failing to recognize that Rawls’s “original position” thought experiment is a heuristic device designed to bolster philosophical imagination and not an alternative to inclusive public deliberation between flesh and blood citizens (Maclure & Weinstock, in press), is insufficient for challenging the distinctiveness of interculturalism. As we have explained, dialogue and contact differ, a point that Modood (2017) himself acknowledges (p. 6–7). However, his discussion of the place of intergroup contact within the theory of multiculturalism is rather short (as compared to the central place given to dialogue). We believe that there is more to be said on the value of contact within a theory of multiculturalism.

Interculturality plays a similar role in dialogue-based political theories and in contact theories: that of reducing racial and ethnocultural prejudice and bigotry. The promotion of face-to-face interactions draws on an insight theorized by Gordon Allport in *The Nature of Prejudice*. The simple idea is that by communicating and interacting with people from different racial and cultural background under conditions of (rough) equality, we are inclined to abandon our prejudices and stereotypes. The idea of a dialogical political theory is akin to this. By incorporating cross-cultural dialogue, we ensure that political principles do not only reflect the dominant culture, that principles remain open to discussion and negotiation and we build trust between communities (Modood, 2017, p. 5).

When discussing the topic of intercultural dialogue, Modood (2017) mentions several of the main contributors to the field of multicultural theory (Benhabib, 2002; Parekh, 2000; Taylor, 1994; Tully, 1995; Young, 1990). What is striking is that one of the main pioneers of this field, Kymlicka, is missing from this picture. This is no surprise, as a normative political philosopher, Kymlicka practices a different style of political theory. He seeks to specify the institutional background conditions--the distribution of rights and duties--that needs to be in place so that each is treated in a fair way. The topic of intercultural contact, as different from intercultural dialogue, finds little examination in his work. He addressed intercultural dialogue mostly through the claim that although multiculturalism needs intercultural citizens capable of interacting constructively despite their different ethnocultural and religious affiliations, there are unresolved tensions between intercultural civic virtues and the multicultural state.^4^ He focused mostly on the educational implications of intercultural citizenship rather than on the importance of promoting face-to-face contact within urban settings (Kymlicka, 2003). However, his account of multiculturalism is not incompatible with the promotion of intercultural interactions. Polyethnic groups in his theory are owed fair terms of integration to the societal cultural of the host society, not autonomous self-government with the capacity to maintain a distinct societal culture (Kymlicka, 1995). The logic of fair integration is to lift obstacles to participation to common societal culture. This, one may argue, will lead to more interactions, not parallel lives. Kymlicka does not link fair terms of integration to Allport’s hypothesis, and does not elaborate on the implications of this for urban planning, which is central in the interculturalism literature (see for instance Landry & Wood, 2007). His more recent engagement with the inter/multi-culturalism debate reasserts that multiculturalism needs to educate citizens to be able to engage in positive interactions with one another and takes for granted Modood and Meer’s view that there is little difference between the theories of interculturalism and multiculturalism, albeit it is currently politically (rhetorically) useful to promote the interculturalism label given the unpopularity of the term ‘multiculturalism’ (Kymlicka, 2016). Yet, although under-theorized in Kymlicka’s work, the role of municipalities in promoting face-to-face contact by designing urban physical spaces conducive to it is not incompatible with his understanding of fair terms of integration. Moreover, as we suggest in the next section, certain propositions of state-based multiculturalism (such as Modood or Kymlicka) are necessary conditions for contact to lead to positive outcomes. This resonates with Kymlicka’s view that intercultural citizenship and the multicultural state are complementary.^5^

### III face to face contact and the state level

One reason why Zapata-Barrero (2017) rejects the multiculturalism policy paradigm is that it is state-centred. Just as, in his view, civic integration focuses exclusively on the duties that the state must enforce, multiculturalism focuses exclusively on the rights that the state must protect. Both paradigms fail to see the role that municipal policies can play in setting up physical spaces conducive to intercultural contacts, which are likely to generate social cohesion, social capital and mutual understanding. We would like to suggest that it is not clear that the promotion of contacts at the local level is likely to lead to those desirable outcomes without support from the state.

Contact, to work, presupposes equality of status. This is recognized by Allport himself, who added several necessary or facilitating conditions to the simple formulation of the contact hypothesis (1954, p., 281; as recognized by Zapata-Barrero on p. 8). He believes that were contact to happen between individual who do not enjoy equal status, it is not likely to undermine intergroup prejudice. In addition to the equality of status condition, Allport adds that, to generate positive outcomes, contact must benefit from institutional support, must be sustained and must happen between people who have common goals.

It is true that local communities and municipalities can do a lot to promote face-to-face contact: they can design enjoyable public spaces – parks, markets, sidewalks, attractive neighbourhoods (Council of Europe, 2008, 2013; Landry & Wood, 2007). However, most of the triggering conditions above-mentioned are items that the state usually provides and secures. Equal-status of all citizens is best promoted when the state avoids symbolically representing some citizens as inferiors, adopts anti-discrimination laws and redistributes wealth more equally. The state is also a key actor in providing institutional support for long-term contact. For instance, public schools are one of the most important places where meaningful intercultural contact can happen. In employment, the public sector is another important place where contact on a daily basis can be maintained. The national level of politics can also be the locus of important common goals when citizens gather around a collective societal project: rebuilding the economy after a crisis, getting rid of corruption, facing an environmental crisis, etc.

Zapata-Barrero (2017) seems to worry that the only thing that the state can do is to use its coercive power to enforce duties and protect rights. This would be an inherent limitation of state-centred multiculturalism. In contrast, interculturalism, focused on the promotion of contact, cannot be compulsory, given the well-known limits of political perfectionism: “We cannot perceive interculturalism as something that should be compulsory, as if it were a perfectionist philosophy. If people do not want to communicate, we cannot force them” (pp. 8–9). Although, we endorse the rejection of political perfectionism in relation to intercultural contact, the allegedly non-compulsory character of interculturalism offers no basis to sharply distinguish this policy paradigm from the multicultural one. To see this, we believe that Zapata-Barrero’s characterization of interculturalism as non-compulsory needs to be unpacked. Four things need to be said. Firstly, a liberal state should obviously not force people to adopt a conception of the good life based on interacting with people from different ethnocultural backgrounds. That a life shaped by cross-cultural friendships and deep understanding of various cultures is superior to a life deeply immersed into one single culture is perhaps a worthy personal ethical ideal, but it cannot be the justification of an enforceable public norm. Secondly, it is not illegitimate to encourage cross-cultural interactions if there is reasonable evidence that this will lead to greater toleration and respect between different ethnocultural groups. Having citizens who can tolerate one another and respect one another’s rights is a political value, not a comprehensive personal ethical ideal. Thirdly, although one can identify public reasons to encourage contact, directly forcing people to interact is not likely to be feasible for pragmatic reasons. Fourth, that being said, we could still maintain that, on the basis of the instrumental value of interactions, public authorities can nudge people into having more intercultural contacts by creating contact zones and shared spaces of face-to-face interactions: “community gardens, libraries, public amenities, festivals and neighbourhood spaces” (p. 14).

However, to conclude from this that interculturalism is not coercive (as opposed to the multicultural or civic integration paradigms) presupposes an unduly narrow conception of state coercion. Indeed, many things that the state and municipalities can do to promote contact is at least indirectly coercive. To promote contact within libraries, park, public squares and public markets and cultural festivals, governments, national or municipal, need funding that they get from mandatory taxation. Moreover, intercultural urban planning decisions are made to shape urban spaces in certain ways. Desirable and justifiable as this may be, such decisions impose one spatial configuration rather than another onto citizens who move through to the cities. Intercultural urban planning is not individualist libertarian *laisser-faire*.

In sum, we can welcome the claim that multiculturalists should think more about the key role of the municipal level of government. This, however, does not mean that a sound policy paradigm dealing with the challenges of post-immigration diversity can do away with the role of the state. This is especially not the case of a paradigm based on the contact hypothesis, as the state has an important role in ensuring the conditions for the success contact between citizens belonging to different cultural groups.

### IV group recognition and intercultural contact

Modood (2017) welcomes the claim that multicultural political theory should incorporate a reflection on the value of face-to-face interaction and the means to better promote them. On the other hand, he also emphasizes the importance of recognizing groups as such. He embraces the commonplace criticism of groupism and essentialism, but thinks that group belonging is still politically salient and that groups can and should be the recipients of certain policies and rights. How well can this recognition of the salience of groups mesh with the promotion of intercultural contact? Would special representation for cultural minorities undermine deliberation with outsiders? Won’t separate ethnoreligious schools prevent pupils to interact with people who do not share their ethnic and religious identity? Won’t cultural festivals only attract members of the culture being celebrated and thus reinforce in-group boundaries? Those are legitimate concerns and perhaps they provide support to Zapata-Barrero’s willingness to do without group-specific treatment and to his reliance on mainstreaming policies.

However, one must keep in mind that group-specific treatment may be necessary to correct patterns of discrimination and injustices that hinder the equal status of minority groups, which is, again, one of the preconditions of the contact hypothesis. Immigrant minorities are vulnerable to specific patterns of exclusion and discrimination that require group specific measures (language acquisition, diploma recognition, anti-racism, ensuring the visibility of minorities in the medias and public offices, accommodation in public institutions, etc.). This implies that mainstreaming cannot be a fundamental overarching principle that rule out all specific treatment targeting immigrant groups.

Moreover, it is not clear that avoiding measures targeting specific minorities is always better at fostering contact. Targeted measures aimed at accommodating minority cultural practices within shared public institutions and at increasing minorities’ presence in the medias and public offices are likely to make diversity more visible and thus to make contact more meaningful and conductive to real engagement with differences. One may think that public funding of minority cultural festivals and cultural organizations will lead to ghettoization and the celebration of diversity at the expense of unity. However, it is through such events and through the work of such organizations that minority cultures become visible to the rest of society and that opportunities for meaningful exchanges are created (Boucher, 2016). Once tangible in this way, differences can be acknowledged, discussed and accepted. Interactions which actively engage with cultural differences may be more likely to lead to positive outcomes than interactions which do not explicitly engage with cultural differences.

Even the most difficult cases of group recognition may hinder contact less than is often thought. For instance, special political representation, in the form of reserved seats in the legislatures or ethnocultural quotas within political parties, does not amount to creating separate ethnic parties and it could perhaps lead to putting diversity issues on the democratic agenda, thus leading to deliberation that engages with minority points of view rather than ignoring it. Separate ethnoreligious schools pose a greater threat to intercultural contact. As we have highlighted, not all multiculturalists are in favour of them (Kymlicka, 2009). Still, we should note that such schools may contain hidden layers of diversity within a distinct group (class, ethnic and racial diversity within a single religious schools) and that in certain geographic areas which do not have a diversified population, common schools are not necessarily more diverse than separate schools.

## Conclusion

In this paper, we have argued that as a civic and liberal political theory, multiculturalism is not a policy paradigm promoting ethnocultural seclusion and the relinquishment of cultural continuity (see Maclure, 2010, pp. 40–41).. Secondly, although the goal of promoting contact is not incompatible with multiculturalism (and is often embraced by multiculturalists), it is conceptually distinct from the notion of intercultural dialogue central in several multicultural theories. Moreover, even though the theorization of face-to-face contacts in urban spaces is neglected in traditional multicultural theories, intercultural contact is only effective when supported by national or state-based policies of multiculturalism. Finally, in order to promote positive intercultural contacts, we cannot abandon group-targeted measures in order to rely exclusively on mainstreaming, although there are some tensions between group recognition and the promotion of intercultural contact.

## Footnotes

1 See also Collett & Petrovic (2014).

2 One could also add that in his liberal theory of multiculturalism, Kymlicka (1995, 2001) is quite comfortable with the view that the culture of the majority is given a privileged status as compared to that of immigrants. To start with, he claims that immigrants cannot hope to recreate their societal cultural in their host society and must integrate it. Also, he maintains that host societies cannot abandon promoting and privileging certain cultural practices. The main example of this is language. Although societies can (and should) avoid having an official religion, they cannot avoid functioning with one particular language and adopting the language of the historical majority is natural. He further claims that several measures of accommodation of immigrant groups (group-specific polyethnic rights) are in fact a form of compensation for the impossibility of having a fully culturally neutral public sphere.

3 We don’t have the space to expand here, but it should be noted that the object of Bouchard’s theory is what Quebec’s interculturalism policy *ought to be* (according to him), not a description of Quebec’s past or current policy of immigrant integration and inclusion. See the references to interculturalism in Government of Quebec (1990) and Government of Quebec (2015).

4 To be more specific, Kymlicka believes that although intercultural citizenship supports the multicultural state and vice-versa, there are unresolved tensions as 1) people are often more interested in gaining cultural understanding of global cultures rather than learning a the cultures of those who share the state with them (Belgian francophone more interested in learning English than Flemish); 2) some ethnocultural groups (conservative religious groups) have isolationist preferences accommodated by the multicultural state but irreconcilable with intercultural citizenship and 3) sometimes the goal of intercultural understanding is unrealistic and what is needed for toleration in the multicultural state is acceptance that others have deeply-held commitments, not a deep mutual cross-cultural understanding on the content of those differences. (see Kymlicka, 2003, pp. 159–165).

5 More specifically on the complementarity between intercultural cities and the multicultural state, see Boucher (2016).
